# Editorial to “Notched P‐wave on digital electrocardiogram predicts the recurrence of atrial fibrillation in patients who have undergone catheter ablation”

**DOI:** 10.1002/joa3.13067

**Published:** 2024-05-23

**Authors:** Satoshi Yanagisawa, Yasuya Inden, Toyoaki Murohara

**Affiliations:** ^1^ Department of Cardiology Nagoya University Graduate School of Medicine Nagoya Japan; ^2^ Department of Advanced Cardiovascular Therapeutics Nagoya University Graduate School of Medicine Nagoya Japan

**Keywords:** atrial fibrillation, catheter ablation, interatrial block, P‐wave, P‐wave morphology

In the current issue of the *Journal of Arrhythmia*, Okuyama et al.^1^ retrospectively investigated the prognostic value of notched P‐wave on electrocardiogram (ECG) automatically evaluated in a specific analyzer system in patients with one or more cardiovascular risk undergoing catheter ablation for atrial fibrillation (AF). Among 100 patients selected from the two previous cohort studies, 28 had recurrence, and the notched P‐wave with a distance of ≥20 ms in lead II was documented in 26 patients. The presence of the notched P‐wave was independently associated with recurrence after ablation in multivariate analysis.

P‐wave morphology assessment on ECG is an old clinical tool; however, it is also essential for the diagnosis and treatment in clinical practice. P‐wave morphology is characterized by several components: (a) the origin of the sinus or atypical rhythm forming the right atrial (RA) depolarization vector; (b) the left atrial (LA) breakthrough and strength of interatrial conduction pathways, which defines the LA depolarization vector; and (c) the size of atrial chambers and structural abnormalities in atrium, requiring time duration of the depolarization process.^2^ An abnormal P‐wave morphology, reported to be associated with AF and ischemic stroke incidence, includes P‐wave duration, advanced interatrial block, P terminal force in lead V1, and P‐wave axis and amplitude.^3^ Specifically, P‐wave morphology in inferior leads can estimate the extent of interatrial block and the conduction capacity of the atrial septal pathways. The P‐wave morphology in lead II consists of the former part activated from the RA and the latter from the LA, with the two components separating as the interatrial block emerges progressively. This partial interatrial block type is thought to be caused by damage in Bachmann's bundle or decreased interatrial impulse propagation in the upper and posterior parts of the interatrial septum and represents a prolonged notch interval between the two components with the valley on ECG, which the authors have tested for prognosis in this study.

One limitation when assessing ECG parameters manually is the variability and inaccuracy of the measurement. Additionally, despite the magnification of the digital recordings, the short duration or amplitude of P‐waves can be difficult to measure. Therefore, automatic calculation using an ECG analyzer system is desirable to resolve the above‐mentioned issues, reducing human effort and unintended variations.^4^ This is because the difference in the notched interval presented in figure 1 in this article is too small to distinguish the distance and components with the valley from the visual appearance of the surface ECG. However, the utility of this analysis may be limited to M‐shape patterns of P‐waves with two positive components, and it is unclear whether this algorithm can be adapted to advanced interatrial blocks presenting with biphasic (positive/negative) or negative P‐wave morphologies in the inferior leads. This kind of abnormal P‐wave represents progressively damaged interatrial conduction, possibly caused by interrupted conduction over Bachmann's bundle and retrograde LA activation via inferior pathways proximity to the coronary sinus ostium, whose activation typically remains until the end. Given that the authors stated in the Methods that no patients presented with negative P‐waves, the study population may consist of patients with mild to moderate damaged conduction and remodeling of the heart, possibly excluding patients with severe remodeling and conduction disturbance.

Another important limitation of the current study is the timing of the ECG assessment the day after catheter ablation for AF. In our experience, published in a recent study,^5^ P‐wave duration significantly decreased by >10 ms from before to just after the ablation, regardless of subsequent reconnection of pulmonary vein (PV)‐LA conduction. We speculated that the isolated area from successful PV isolation may form a substantial component of the P‐wave. Furthermore, the notched P‐wave morphology remarkably changed following ablation as presented with the delayed or new appearance notch, possibly associated with the reconnection and conduction delay of the PV potential. Thus, assessing P‐wave morphology before rather than after the ablation is strongly recommended. Perhaps the 40 patients included in the current study had persistent AF, and the opportunity to evaluate P‐wave during the sinus rhythm may be limited before the procedure. However, even after cardioversion, P‐waves can be affected by autonomic nervous tone, heart rate, and drug administration, making it difficult to reduce potential effects completely.^2^

Despite the aforementioned limitations, this study suggests an important perspective that characterizes early electrical remodeling prior to mechanical and structural atrial remodeling, leading to the stratification of patients undergoing catheter ablation for AF. This was supported by a multivariable analysis showing that the notched P‐wave, but not the LA volume index, remained an independent predictor of recurrence. In recent studies, there is accumulative evidence regarding advanced interatrial block and risk of recurrence after catheter ablation AF. A direct explanation for the link between these two features remains unclear; however, the delayed conduction between the RA and LA provokes interatrial dyssynchrony, leading to electrical heterogeneity, impaired mechanical function, and development of atrial fibrosis in the LA, which may lead to decreased success rates following ablation. Determining whether P‐wave abnormalities are caused by atrial enlargement or interatrial conduction delay may be difficult; in addition, previous reports have not addressed the association between atrial dilatation in imaging studies and the electrical abnormality of P‐wave.^3^ Assessment of P‐wave morphology at an early stage after ablation using a universal method with low variability may be helpful in stratifying patients at risk of recurrence and in planning a careful follow‐up after ablation beforehand. To validate the predictive power of the notched P‐wave assessed preoperatively after catheter ablation for AF, future prospective studies with large sample sizes are needed.

## CONFLICT OF INTEREST STATEMENT

Dr. Yanagisawa is affiliated with a department‐sponsored by Medtronic, Japan. Other authors have no conflicts of interest.

## FUNDING INFORMATION

None.
